# Alterations in bacterial community dynamics from noncancerous to Gastric cancer

**DOI:** 10.3389/fmicb.2023.1138928

**Published:** 2023-03-09

**Authors:** Xuan Peng, Siqi Yao, Jing Huang, Yiming Zhao, Hao Chen, Liyu Chen, Zheng Yu

**Affiliations:** ^1^Department of Microbiology, School of Basic Medical Science, Central South University, Changsha, China; ^2^Department of Medical Parasitology, School of Basic Medical Science, Central South University, Changsha, China

**Keywords:** gastric carcinogenesis, microbiome, gastric cancer, microbial interactions, *Lactobacillus*

## Abstract

Gastric microbiome has been shown to contribute to gastric carcinogenesis, understanding how alterations in gastric microbiome is helpful to the prevention and treatment of gastric cancer (GC). However, few studies have focused on the change of microbiome during the gastric carcinogenesis. In this study, the microbiome of gastric juice samples from healthy control (HC), gastric precancerous lesions (GPL) and gastric cancer (GC) was investigated by 16S rRNA gene sequencing. Our results showed that the alpha diversity of patients with GC was significantly lower than other groups. Compared to other groups, some genera in GC group were shown to be up-regulated (e.g., *Lautropia* and *Lactobacillus*) and down-regulated (e.g., *Peptostreptococcus* and *Parvimonas*). More importantly, the emergence of *Lactobacillus* was closely related to the occurrence and development of GC. Moreover, the microbial interactions and networks in GPL exhibited higher connectivity, complexity and lower clustering property, while GC showed the opposite trend. Taken together, we suggest that changes in the gastric microbiome are associated with GC and perform a key function in maintaining the tumor microenvironment. Therefore, our findings will provide new ideas and references for the treatment of GC.

## Introduction

Gastric cancer (GC) is the fifth most frequently diagnosed cancer and the fourth-leading cause of cancer-related deaths worldwide (Sung et al., 2021). Although the incidence and mortality rates are declining with the advancement of therapeutic, GC is still a global medical burden (Thrift and El-Serag, 2020). Therefore, it is necessary to understand the mechanism of GC to better treat and prevent the occurrence of this disease. The development of GC can be described as a series of sequential stages: from chronic superficial gastritis, chronic atrophic gastritis, intestinal metaplasia, dysplasia to GC (Correa, 1992). The microbiome is involved in the development of GC and the composition of gastric microorganisms varies in individuals at different stages of the stomach (Stewart et al., 2020). Previous studies have suggested that many factors are associated with the development of GC. In addition to genetic predisposition, environmental factors including microbial community interactions have been shown to contribute to GC (Kwon et al., 2021).

Microbiome is considered a significant component of the tumor microenvironment. The human gastrointestinal tract contains a large number of microbiomes, including bacteria, fungi and viruses. *Helicobacter pylori* (HP) infection is a fundamental factor for gastric lesions (Vinasco et al., 2019). However, the presence of HP is not the only factor involved in gastric carcinogenesis, as only a minority of HP infected individuals develop GC, implying that other factors also perform a key function (Bjorkholm et al., 2003). Naturally, more attention has been paid to the function of gastric microbiome diversity in the development of GC. Experiments in the insulin-gastrin transgenic (INS-GAS) mouse model have demonstrated that the synergy among bacterial communities promotes gastric tumorigenesis (Lertpiriyapong et al., 2014; Whary et al., 2014). Later, gastric microbiome was also found to produce carcinogenic N-nitroso compounds and secondary amines by metabolizing food, which strengthened the role of gastric microbiome in the development of GC (Tseng et al., 2016). A study found the relative abundance and diversity of microbiome in the GC group decreased compared to patients with chronic gastritis (Ferreira et al., 2018). In addition, changes in gastric microbiome in patients with GC have been detected by using 16S rRNA gene sequencing in several studies recently, and gastric carcinogenesis was revealed to be associated with microbial dysbiosis (Chen et al., 2019; Gantuya et al., 2020; Zhang et al., 2021). Together, these findings highlighted the potential role of microbial community structure diversity in the development of GC.

Some studies have been devoted to revealing the differences in the microbial composition from noncancerous to GC. However, there is no consensus on the microbiome changes in the pathological stages of GC. In this study, gastric juice samples from patients with healthy control (HC) gastric precancerous lesions (GPL) and GC were collected, aiming to explore the differential distribution profile of microbiome in different stages of gastric lesions by using 16S rRNA gene sequencing. Our findings will help explore the role of gastric microbiome in carcinogenesis.

## Materials and methods

### Study subjects and sample collection

Total 60 participants, including 22 of HC, 22 of GPL and 16 of GC, were recruited at the Xiangya Hospital, Changsha, Hunan, China from October 2015 to November 2016. The demographic characteristics of participants were shown in Table 1. Among them, the HC group mainly consisted of patients with gastritis. Gastritis and gastric precancerous lesions patients were diagnosed by the gastrointestinal endoscopes Department of Xiangya Hospital according to the clinical practices (Chinese Medical Association, 2006; Medical Secretary, M.O.H., People's Republic of China, 2012). In addition, GPL refers to a kind of pathological change of gastric mucosa liable to cancerization. The inclusion criteria of GPL were atrophic gastritis, intestinal metaplasia or adenomatous polyp. The study was approved by the independent Ethics Committee of Xiangya Hospital of Central South University following the ethical guidelines of the Declaration of Helsinki (No. 038, 2015). Participation was voluntary and written informed consent was obtained from all participants.

**Table 1:** Characteristics of study subjects.

Variables	HC (*n* = 22)	GPL (*n* = 22)	GC (*n* = 16)	*p*-value
Age [year, median(range)]	49.5 (32–60)	48.5 (32–59)	59.5 (44–81)	0.00008
Gender, *n* (%)				0.516
Female	9 (40.9%)	12 (54.5%)	6 (37.5%)	
Male	13 (59.1%)	10 (45.5%)	10 (62.5%)	
Smoking history, *n* (%)				0.393
Yes	5 (22.7%)	4 (18.2%)	1 (6.3%)	
No	17 (77.3%)	18 (81.8%)	15 (93.8%)	
Drinking history, *n* (%)				0.216
Yes	2 (9.1%)	5 (22.7%)	0 (0%)	
No	20 (90.9%)	17 (77.3%)	16 (100%)	

Exclusion criteria were as follows: age under 18 years; the presence of a serious illness such as severe cardiopulmonary, renal, or metabolic diseases; prior medication history of antibiotics, acid drugs (proton pump inhibitors and H_2_ receptor antagonists), probiotics, or anti-inflammatory drugs (aspirin, nonsteroidal and steroids) for past one month; A large amount of alcohol and smoke for past a month. All patients were subject to endoscopy or biopsy.

The patients were treated according to clinical requirements and then undergo gastrointestinal endoscopy. Approximately, 10 mL of gastric juice was collected from the gastroscope with sterile syringe, then filtered by the double sterile gauze to remove food debris and stored in sterile 10–15 mL tubes. The tubes were kept at 0°C for no more than 12 h prior to DNA isolation. DNA isolation of the bacterial sediments was performed using the QIAamp^®^ FAST DNA Stool Mini Kit (QIAGEN) according to the manufacturer’s protocol after centrifuging at 12,000 rpm, 4°C, for 10 min.

### Polymerase chain reaction and high-throughput sequencing of 16S rRNA gene

The V4 region of the 16S rRNA gene was amplified by polymerase chain reaction (PCR) with a universal forward primer and a unique barcoded fusion reverse primer (515\u00B0F: 5′-gtgccagcmgccgcggtaa-3′ and 806 R: 5′-ggactachvgggtwtctaat-3′). PCR was performed using 30 ng of genome DNA, V4 Dual-index Fusion PCR Primer Cocktail and PCR Master Mix (NEB Phusion High-Fidelity PCR Master Mix) for per reaction. The melting temperature is 56°C and PCR cycle is 30. The PCR products were purified with AmpureXP beads (AGENCOURT) to remove the unspecific products. The library was quantitated by determination of the average molecule length using the Agilent 2100 bioanalyzer instrument (Agilent DNA 1000 Reagents) and quantification of the library by real-time quantitative PCR (EvaGreenTM). Then, the qualified libraries were sequenced by the way of pair-end on the Illumina MiSeq System with the sequencing strategy PE250 (MiSeq Reagent Kit) by Beijing Genomics Institute (BGI, Wuhan, China).

After sequencing, the reads were de-multiplexed into samples according to the barcodes. Fitter sequences were imported to the Quantitative Insights into Microbial Ecology (QIIME2, 2022.2)^1^ (Bolyen et al., 2019). The raw data were filtered to eliminate the adapter pollution and low-quality reads to obtain clean reads, and then use DADA2 denoise-paired to dereplicate sequence data and create a feature table and feature representative sequences (Callahan et al., 2016). Taxonomic classifiers use plugin feature-classifier, classify-consensus-blast, based on Silva 138 reference sequence (MD5: a914837bc3f8964b156a9653e2420d22) and taxonomy files (MD5: e2c40ae4c60cbf75e24312bb24652f2c) (Camacho et al., 2009; Bokulich et al., 2018; Robeson et al., 2021). Use plugin taxa removal of non-bacterial sequences and mitochondrial chloroplast contamination.

### Statistical analysis

All statistical analyses were performed using the R V4.1.2 environment (R Core Team, 2022). No special instructions, the statistical results were visualized using the “ggplot2” package (Wickham, 2009). Alpha diversity was measured using the function diversity in the package “Vegan” based on a flat taxonomy table. Gini-Simpson diversity index was obtained by subtracting the value of the classical Simpson index from 1. Beta diversity was compared using principal coordinate analysis (PCoA). Bacterial community composition across all samples based on Bray-Curtis distances. Redundancy analysis (RDA) was also conducted using Vegan (Lai et al., 2022). Package rdacca.hp. was used to obtain conditional effect base on Hierarchical Partitioning (Oksanen et al., 2022). Beta diversity across sample groups was compared by PERMANOVA with permutations of 999. ANOSIM was chosen to test for significance between groups (Wang et al., 2008), *R* > 0, *p* < 0.05 was considered significant. The DEseq2 package was used to analyze abundance difference and Marker genus (Topper et al., 2017). The differential expression matrix and the *p*-value matrix of species composition were obtained through the function DESeqDataSetFromMatrix. The significant level was *p* < 0.05 and the absolute FoldChange value greater than 2. The coexistence network of three groups was established based on Spearman correlation matrix and corrected *p*-value matrix using the igraph package; the Benjamini and Hochberg false discovery rate (FDR) were used to correct the *p* value; modules were divided according to the high intra-module connectivity and the low inter-module connectivity; Spearman correlation coefficient and corrected *p* values were 0.8 and 0.05, respectively, (Yuan et al., 2021). Gephi software^2^ is used to calculate the network topological properties and the hub network. Among them, clustering coefficient is a measure of the degree of clustering property of microorganism. Abundance ratio greater than 0.05 genus abundance heatmap was created using the pheatmap package, and the abundance information was transformed by adding one, then taking the logarithm of ten (Kolde, 2019). Phylum level Manhattan plot computed using edgeR package based on taxonomy information (Robinson et al., 2010).

In addition, biomarkers of sample groups were discovered by Linear Discriminant Analysis (LDA) Effect Size (LEfSe)^3^ (Segata et al., 2011). The strategy for multi-class analysis was set one-against-all, and the threshold on the logarithmic LDA score for discriminative features was set to 2.0.

## Results

### Clinic characteristics of study subjects

The demographic characteristics of the HC, GPL, and GC groups are shown in Table 1. There is no difference in gender, smoking history, and drinking history, but difference in median age was detected (*p* < 0.05) among the three groups. On the one hand, RDA analysis showed that gender and age of all participants accounted for only 3.37% of the composition and distribution of microorganisms in gastric juice (Supplementary Figure S1A). On the other hand, the conditional effect of age on microorganisms in gastric juice is 0.70% (Supplementary Figure S1B). Moreover, the clinical data of the GC group population were shown in Table 1. The condition of patients with HP infection was shown in Supplementary Tables S1, S2.

### The diversity of gastric juice microbiome

**Figure 1:**
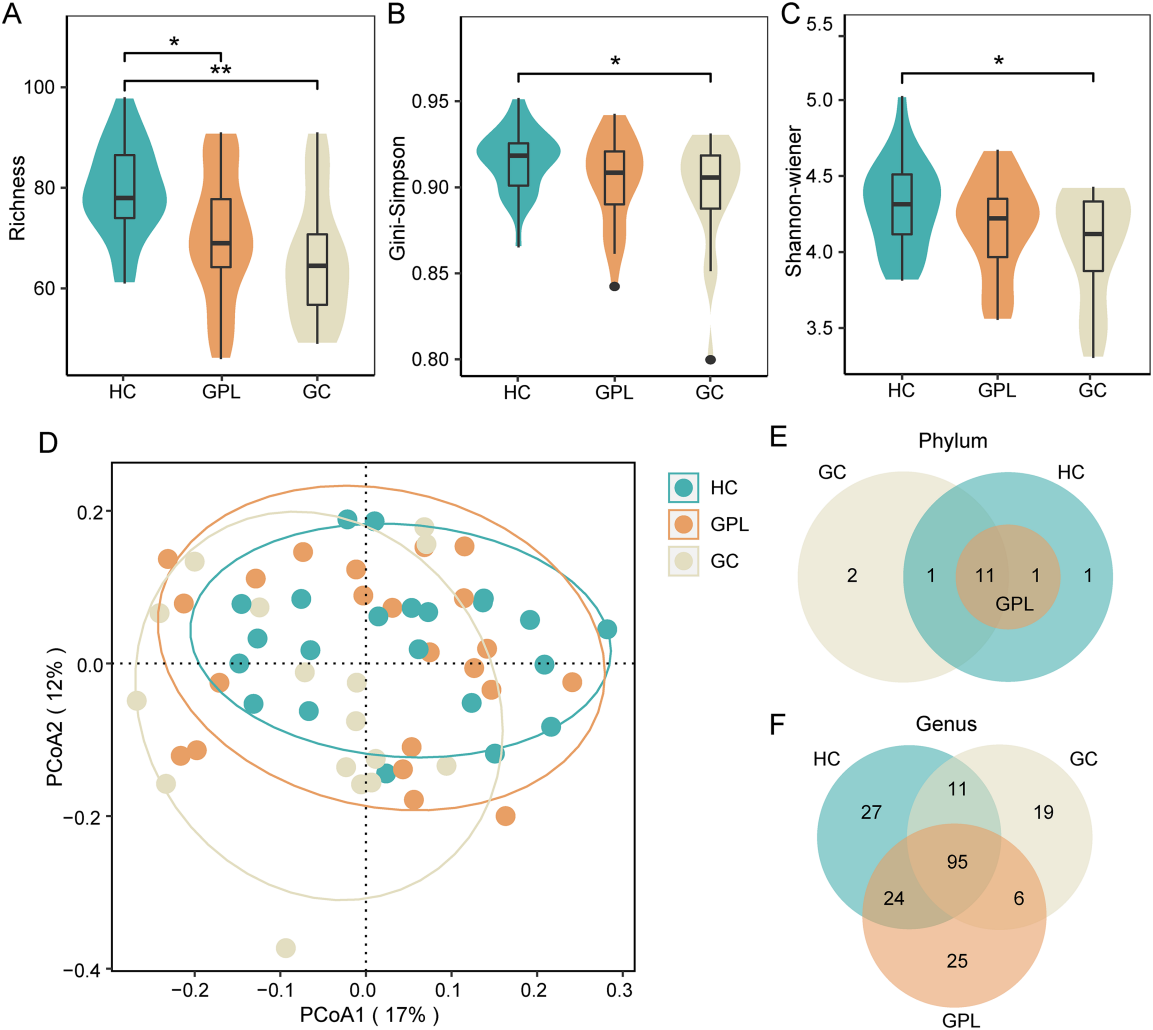
The microbial diversity of microbial communities. Alpha diversity index analysis richness **(A)**, Gini-Simpson **(B)**, and shannon-wiener **(C)** (kruskal–wallis test, *p < 0.05, **p < 0.01). **(D)** Beta diversity PCoA analysis (ANOSIM R = 0.077, *p* < 0.05), the ellipse contains 85% of the samples in each group. The difference of composition in phylum **(E)**, genus **(F)** level between three groups. HC, healthy control; GPL, gastric precancerous lesions; GC, gastric cancer.

Diversity rarefaction curves of all sample species tended to be parallel to the *X*-axis, indicating that there is a significant difference (Supplementary Figure S2). The alpha diversity indices Richness (Figure 1A), Gini-Simpson (Figure 1B) and Shannon Wiener (Figure 1C) decreased gradually with disease progression among HC, GPL, and GC groups, there are significant differences among Richness groups (*p* < 0.05). Compared by using principal coordinate analysis (PCoA), and the ANOSIM test (*R* = 0.077, *p* < 0.05), beta diversity demonstrated significant differences among groups. The PCoA results showed that clustered within groups with a smaller area in the HC group than GPL and GC groups, where the Bray-Curtis distances between HC and GPL were closer than HC and GC groups (Figure 1D; Supplementary Figure S3). The difference between groups was greater than that within groups, implying a significant difference in diversity among HC, GPL and GC groups (Figure 1D).

### The composition dynamics of gastric juice microbiome

#### Gastric juice microbiome composition

**Figure 2:**
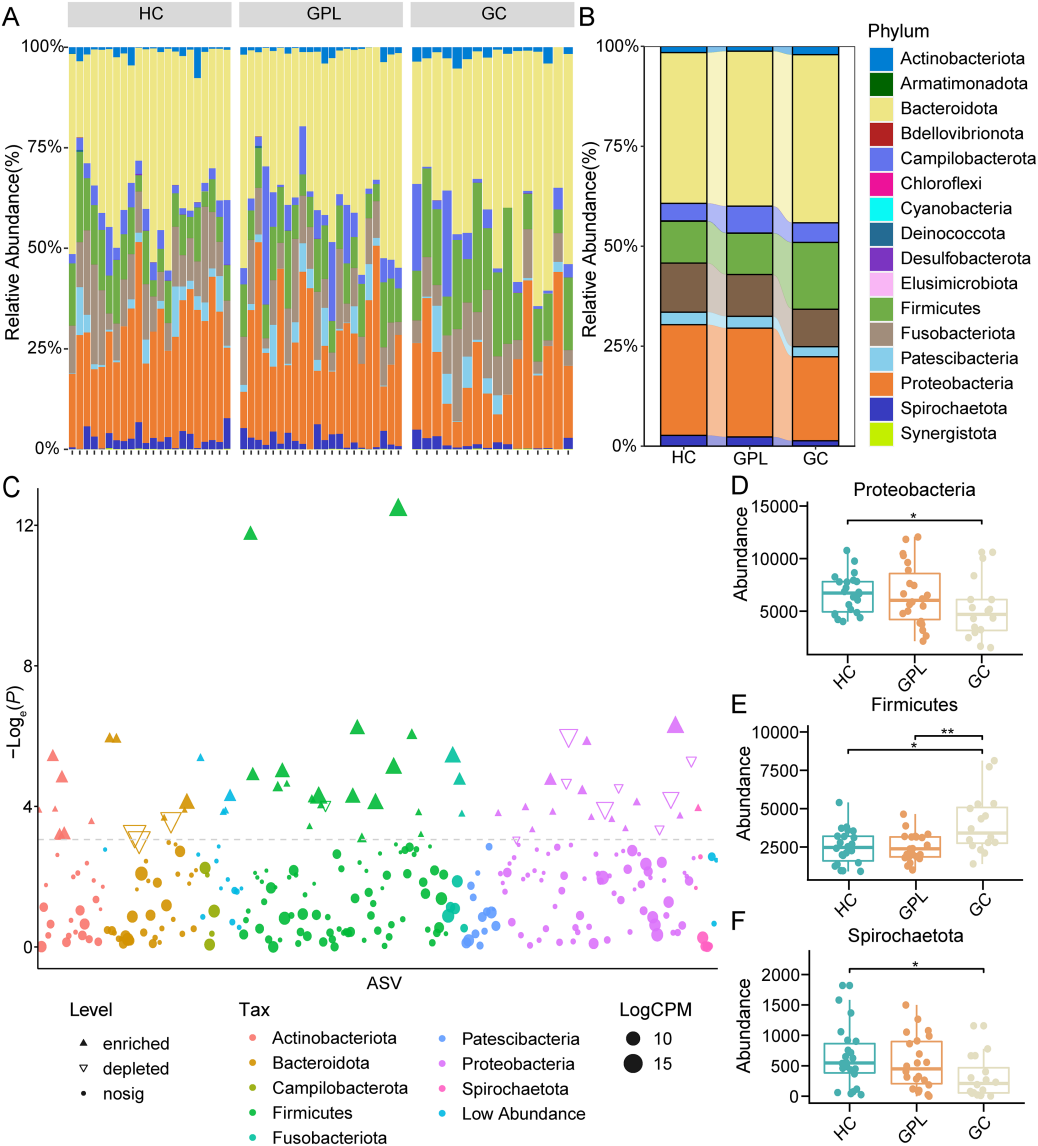
The relative abundances of taxonomy at the phylum level. **(A)** The relative abundances of taxonomy at the phylum level in all samples (60). **(B)** The relative abundances in phylum level in three groups. **(C)** Demonstration of precancerous lesions gastric cancer (GPL/GC) versus bacterial group with healthy control (HC). *X*-axis for ASVs, alphabetically ordered by taxonomic phylum level; *Y*-axis *p* value values for the comparison of the two groups, taken as log_e_ (*P*), i.e., natural logarithmic transformation; the size of the nodes in the graph represents the relative abundance of that ASV, taken as log_2_ (CPM), the logarithm of 2; CPM is an abbreviation for count per million, which being fractions of a million; different nodes colors represent different phylum; the shape of the nodes in the graph marks the type of its change, whether it is up-regulated enriched (positive solid triangle), down-regulated depleted (inverted hollow triangle), or no significant difference change nosig (solid nodes). Differences abundance at the phylum level among groups (Wilcox. test, * *p* < 0.05, ** *p* < 0.01), Proteobacteria **(D)**, Firmicutes **(E)**, Spirochaetota **(F)**.

There were commonalities and differences in bacterial composition among the HC, GPL and the GC group. At the phylum level, 14 phyla were in the HC and GC group while 13 phyla were in the GPL group (Figure 1E). At the genus level, 157 genera were in the HC group, 150 in the GPL group, 131 in the GC group, only 95 genera common to all groups (Figure 1F). The differences in composition at the genus level among the three groups were greater than those at the phylum level. To clarify the bacterial differences among the groups, we compared the differentially abundant bacteria among the three groups at the phylum and the genus level, respectively. At the phylum level, the results indicated that the composition of the three groups was similar, but the relative abundance of components varied (Figures 2A,B). We analyzed the bacterial alterations between the GPL, GC, and HC groups. Comparison with the HC group, the decreased abundance of bacterial species was greater than the increased abundance in the GPL and GC groups (Figure 2C). Similar patterns of variation were also found in the comparison of GC and GPL groups (Supplementary Figure S4). Further analysis of bacterial abundance differences at the phylum level showed the same results. With the course of gastric disease intensified (HC, GPL, and GC), phylum Proteobacteria (Figure 2D) and Spirochaetota (Figure 2F) showed a significant down-regulation of gastric juice microbiome, while Firmicutes phylum (Figure 2E) showed a significant up-regulation (*p* < 0.05). Genus level abundance heatmap showed the distribution of abundance at the genus level for each sample. *Prevotella*, *Alloprevotella*, *Haemophilus*, *Neisseria*, *Fusobacterium* were the high-abundance genus (Supplementary Figure S5).

#### Biomarkers of sample groups discovered by LEfSe, DESeq2, and Metastats

**Figure 3:**
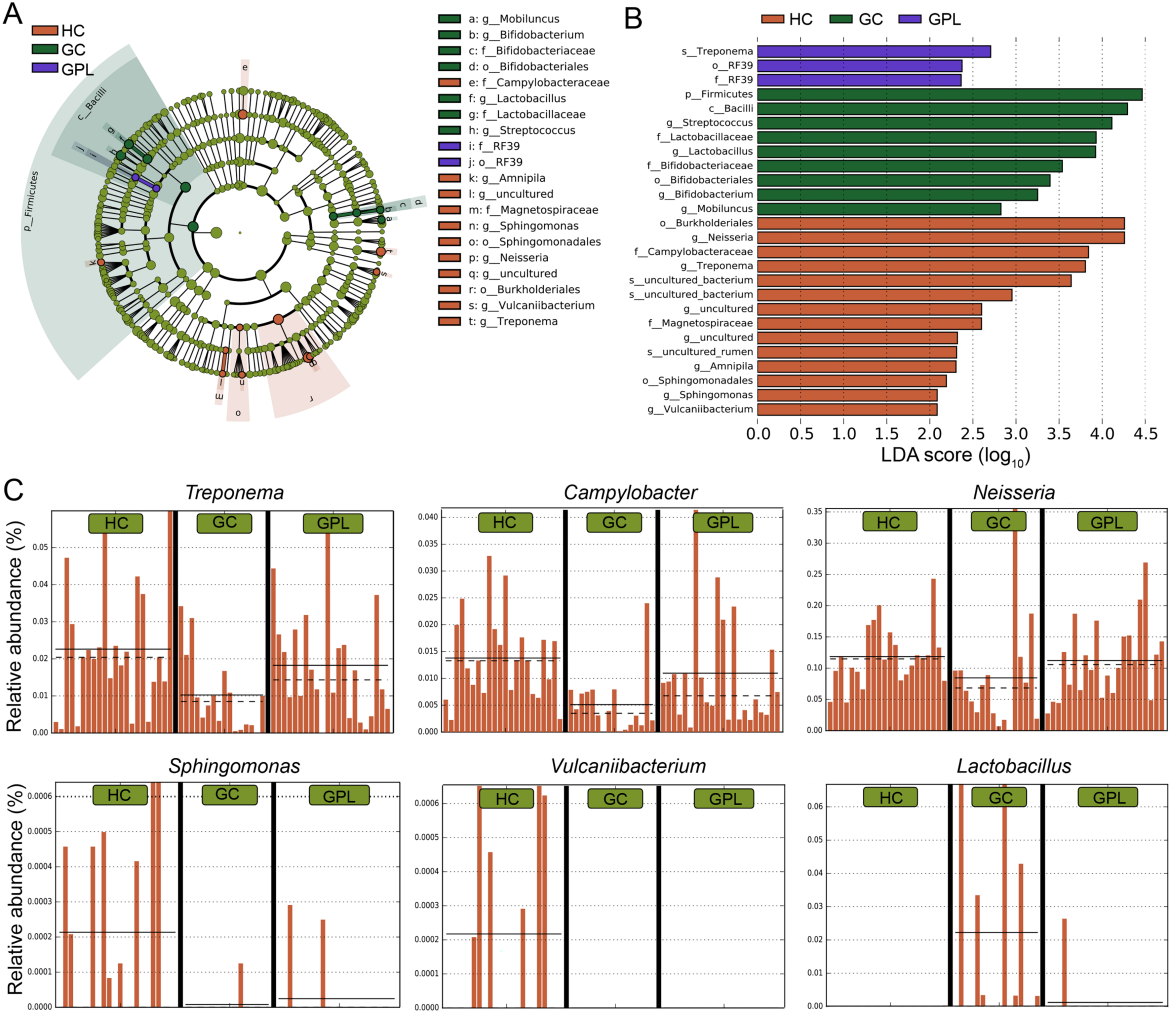
LEfSe analysis of taxonomy with significant differences in abundance among groups. **(A)** Evolutionary branching diagram. The circles radiating from the inside to the outside represent taxonomic levels from the phylum to the genus. Each small circle at different taxonomic levels represents a taxon at that level, and the diameter size of the small circles is proportional to the relative abundance size; species without significant differences are uniformly colored in chartreuse, and the difference species Biomarker follows the group for coloring, red nodes indicate microbial taxa that play an important role in the HC group, green nodes indicate microbial taxa that play an important role in the GC group, purple nodes indicate microbial taxa that play an important role in the GPL group. The names of the species indicated by letters in the figure are shown in the legend on the right. **(B)** Histogram of LDA value, taxon with significantly different abundances in different groups are shown, and the length of the bar graph represents the effect size of the significantly. **(C)** Comparison of the abundance of biomarkers in each sample among HC, GPL and GC groups. HC, healthy control; GPL, gastric precancerous lesions; GC, gastric cancer.

**Figure 4:**
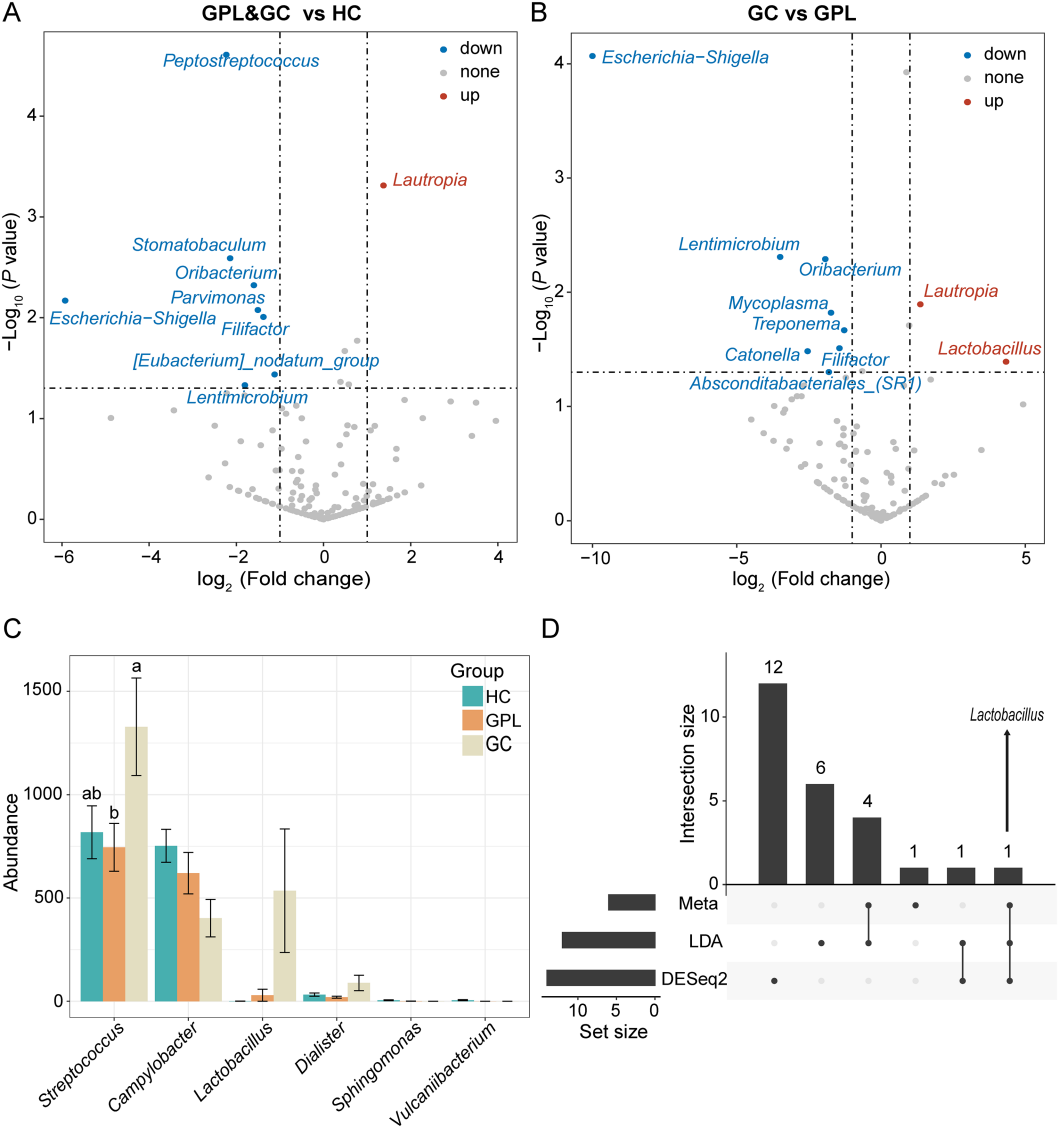
Differential abundance analysis at the species level. The volcano shows significantly up-regulated and down-regulated in the gastric precancerous lesions (GPL) and gastric cancer (GC) group compared with the healthy control (HC) group **(A)**; GC compared with the GPL group **(B)**. The color of the nodes in the volcano marks the type of its change, blue indicates down-regulation, red indicates up-regulation, and gray indicates no significant difference between groups. **(C)** Differential abundance analysis at the genus level base on meta, LDA, DESeq2 and hub genus in co-occurrence network. The different relative abundance among three groups based on metastats. Significant differences among groups are indicated by alphabetic letters above the bars, determined by multiple comparison LSD-T test (*p* < 0.05). **(D)** This upset diagram shows the biomarkers found based on Metastats, LDA and DESeq2.

To better elucidate the gastric juice microbial biomarkers among the HC, GPL, GC groups, we used three methods to analyze the markers. The results of LEfSe showed that the identified intergroup biomarker genera echoed the previous intergroup differences at the phylum level (Figure 3A), and *Treponema*, *Campylobacter*, *Neisseria*, *Sphingomonas*, *Vulcaniibacterium*, and *Lactobacillus* were biomarker genera (Figure 3B). Furthermore, GC and GPL showed decreased abundance of the genera *Treponema*, *Campylobacter*, and *Neisseria*, while *Lactobacillus* abundance increased compared to the HC group. Among them, genera *Vulcaniibacterium*, *Sphingomonas* were commonly found in the HC group but largely undetectable in the GC and GPL groups (Figure 3C). *Lautropia* was up-regulated and *Escherichia-Shigella* was down-regulated in both GC and GPL compared to the HC group, and the same result was observed in the GC group compared to the GPL group (Figures 4A,B). Compared with the GPL group, the abundance of *Mycoplasma*, *Treponema* was down-regulated in the GC group. The metastatic showed the GC group had significantly higher abundance in *Streptococcus* and *Lactobacillus* compared with the GPL and HC (Figure 4C). To further compare the commonalities and differences of the biomarkers discovered by several methods, we plotted the upsets. Interestingly, we found the biomarkers jointly identified based on LEfSe, DESeq2 and Metastats analysis methods have an intersection, which is *Lactobacillus* (Figure 4D).

### The microbial interactions and networks between gastric juice microbiome

**Figure 5:**
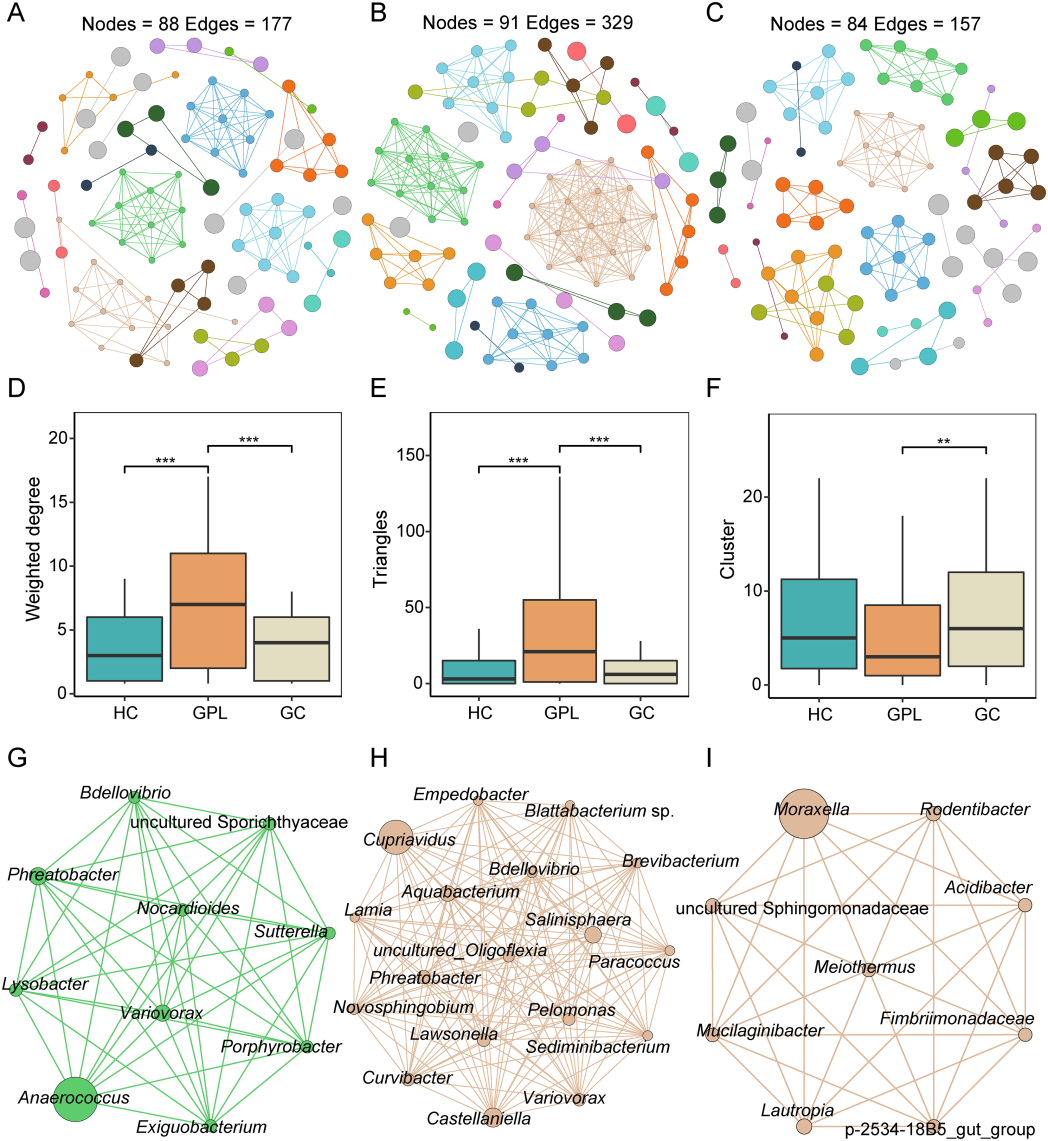
Co-occurrence network in three groups. **(A)** healthy control (HC) network, **(B)** gastric precancerous lesions (GPL) network and **(C)** gastric cancer (GC) network. Comparison of network topology properties among groups, weighted degree **(D)**, triangles **(E)**, cluster **(F)**. Wilcoxon test, **p < 0.01; ***p < 0.001. Co-occurrence hub network, HC **(G)**, GPL **(H)**, GC **(I)**.

We performed a network co-occurrence analysis to unravel the relationships among microorganisms. With the same network construction parameters, the HC group network (Figure 5A) had 88 nodes and 177 edges, the GPL group (Figure 5B) had 91 nodes and 329 edges, while 84 nodes and 157 edges in GC group network (Figure 5C). In addition, HC group and GC groups are all clustered into 23 modules, while GPL group has only 19 modules (Table S3). The results suggested that microbial networks were composed of tightly connected nodes and formed a kind of “small-world” topology (Supplementary Figure S6; Supplementary Table S3). Compared with the topological properties of the random network with the same number of nodes and edges (Supplementary Figure S6; Supplementary Table S4), the network of the HC, GPL and GC groups exhibited a scale-free characteristic (*p* < 0.001, Supplementary Figure S7), indicating that the network structure was non-random. Correspondingly, we also analyzed the network properties for each group of networks. The average degree of the HC and GC groups were 3.078 and 4.023, which were lower than that of the GPL group (7.231, *p* < 0.001, Figure 5D), and the number of sides forming triangles was also lower (*p* < 0.001, Figure 5E). This suggests that total connectivity and complexity between gastric juice microbiome was higher in the GPL group than in the HC and GC group. The network Cluster of the GC group was significantly higher than that of the GPL group (*p* < 0.01, Figure 5F). These results manifested that the average “clustering property” of the whole network between gastric microorganisms in the GPL group was lower than that in the HC and GC groups. To understand each network in three groups deeply, we extracted the microbial hub network. Among the three groups of hub networks, *Anaerococcus* was the highest abundance in the HC group (Figure 5G), *Cupriavidus* was the highest abundant in the GPL group (Figure 5H), and in the GC group hub network, *Moraxella* was the highest abundant genus (Figure 5I). Overall, the GPL group hub network has the highest number of nodes and the highest agglomeration. The above findings can be concluded that there are differences in the gastric juice microorganism interaction network among the HC, GPL, and GC groups. Compared with the HC and GC groups, the GPL group network has the highest connectivity and complexity and the lowest clustering property.

## Discussion

Gastric juice as samples were investigated in the present study, which was different from other studies using gastric mucosa (Yu et al., 2017; Ferreira et al., 2018; Png et al., 2022). Compared to gastric mucosa, gastric juice had two advantages in the study of gastric microbiota its homogeneity and noninvasiveness.

Previous studies have demonstrated that the gastric microbiome structure changes during carcinogenesis (Lam et al., 2017). It has been thought that HP was closely related to the development of GC, but the results of the available studies indicate that the risk of GC was not proportional to the degree of HP infection (Diaz et al., 2018). HP penetrates the gastric mucosa through flagella and mainly adheres to the surface of gastric epithelial cells. Therefore, other microbiome in gastric juice may be of greater importance for GC development (Hessey et al., 1990; Ruch and Engel, 2017). Our study pointed out that the gastric microbial composition and network of patients with GC have changed compared to the patients with HC and GPL. We identified the differences in the gastric microbiome among GC group and other groups, explored potential correlations, and also revealed the interaction between the gastric microbiome. The alpha diversity of GC group was significantly lower than other groups, and the alpha diversity indexes decreased gradually with the progression of gastric lesions. The microbial composition of the patients with GC gradually deviates from other groups. In addition, in the microbial interactions of the three groups, we found that networks of the GPL group exhibited higher connectivity, complexity and lower clustering property, which in the GC group showed the opposite.

The occurrence of GC starts from superficial gastritis, but the key factors promoting the development of GC are still unclear (Correa, 1992; Li et al., 2022). The microbial community is generally recognized as an important biological factor in the development of GC. Previous studies have shown that the structure of gastric microbial diversity was constantly changing in the process of gastric lesions (Wang et al., 2018). In the case of GC patients, the microbial community diversity was quite different from other groups (Coker et al., 2018). Our results were consistent with these showed that the alpha diversity of patients with GC was significantly lower than other groups. In addition, the PCoA analysis of the microbiome composition revealed that there were significant differences in the community structure between the GC group and other groups. This is in keeping with previous findings that the composition of the microbial community changed as the stomach disease gradually deepened (Wang et al., 2018). Therefore, our findings suggested that changes in microbial diversity were critical to the development of GC. The dysregulation of the gastric microbial community may increase the likelihood of developing cancer. Focusing on microbial diversity contributes to a better understanding of GC course and supports GC prevention and treatment.

*Helicobacter pylori* is the earliest microorganism reported to be associated with GC. Apart from HP, there are many other microbiome in the gastric, and their interactions are related to the development of GC (Duan et al., 2022). A Portuguese study showed that bacteria such as *Streptococcus*, *Prevotella*, *Clostridium*, and *Lactobacillus* were significantly increased in GC patients compared with patients with chronic gastritis (Ferreira et al., 2018). In addition, a recent animal study showed that the increasing of *Lactobacillu*s abundance contributed to the development of GC (Dai et al., 2021). In this study, analysis of group differences at the phylum level showed that while the abundance of Firmicutes increased in the GC group, Proteobacteria, and Treponema decreased. To search for potential taxonomies that may be associated with carcinogenesis, we used different methods to identify the most significantly correlated taxa among the three groups. Through LEfSe analysis, we found that *Treponema*, *Campylobacter*, *Vulcaniibacterium* and *Neisseria* were increased in HC group at the genus level, while *Lactobacillus* and *Streptococcus* were significantly enriched in GC group. *Neisseria* is symbiosis bacteria in the mouth and esophagus, but the translocation and expansion of oral bacteria may be involved in the development of inflammation (Han and Wang, 2013). *Campylobacter* is closely related to HP, and despite a normal oral bacteria, numerous studies have found that its high transcriptional activity in gastric acid verified its role as a potential pathogen in the gastric (von Rosenvinge et al., 2013; Liu et al., 2018; Cui et al., 2019). Its ability to cause tissue damage and disease has been attributed to the production of virulence factors, leading to changes in epithelial permeability and local tissue destruction (Istivan and Coloe, 2006; Hess et al., 2012). Although there is no direct evidence that the occurrence of gastritis is associated with Campylobacter infection, chronic pathological changes ultimately result from the adhesion and colonization of *Campylobacter*. The presence of oral microbiome in the HC group suggested that our oral microbiome gradually play a cautionary role in the early stages of gastric lesions. The oral cavity severs as the beginning of the digestive system, its microbiome affects the health of the digestive system. *Vulcaniibacterium*, a moderate thermophilic bacterium (Yu et al., 2013; Niu et al., 2020), was first found in the stomach, suggesting that it may be closely related to the occurrence of gastritis. Meanwhile, we also observed enrichment of *Streptococcus* in GC group, which was consistent with previous findings (Sun et al., 2018; Huang et al., 2021). The abundance of *Streptococcus* has also been reported in several types of cancer, such as colorectal adenocarcinoma (Abdulamir et al., 2011). Taken together, the results suggest that *Streptococcus* may be involved in gastric carcinogenesis. More attention can be paid to the relationship between this bacteria and GC in the future.

Then, we further observed that compared with the HC group by differential abundance analysis. The up-regulated bacteria in cancer patients are *Rotobacter*, while down-regulated bacteria include *Streptococcus pepticus* and *Micromonas*, which are common oral microflora. *Lautropia* has been reported to be more abundant in patients with periodontal disease (Papapanou et al., 2020). It is also the predominant microorganism isolated from the sputum of cystic fibrosis patients (Ben Dekhil et al., 1997) and the oral cavity of children infected with human immunodeficiency virus (Rossmann et al., 1998). However, the possible pathogenic mechanism of the bacteria is still unclear. Our study found that this bacteria is up-regulated in GC patients, and its pathogenic mechanism may serve as a key target for future research on the relationship between oral bacteria and human health. In addition, a study on salivary microbiome found that *Parvimonas* was shown to be inversely associated with the development of GC (Huang et al., 2021), the bacteria were also found in CRC patients (Nakatsu et al., 2015). In the comparison between GPL and GC, we found that *Lactobacillus* also showed up-regulation while *Mycoplasma* and *Treponema* were down-regulated. First, *Mycoplasma* in gastric juice is down-regulated, which may be due to the ability of its lipoprotein P37 periplasmic transport system to promote cell motility and invasion (Gong et al., 2008; Gomersall et al., 2015). It allows *Mycoplasma* to colonize on the gastric mucosa by breaking through the mucosal barrier of gastric juice, eventually leading to *Mycoplasma* decreased. Secondly, *Treponema* can weaken MMPs’ regulation to tumor cell tissue invasion and exocytosis because the mucosal invasiveness of its major virulence factor chymotrypsin-like protease (Td-CTLP) and the hydrolysis of host-derived matrix metalloproteinases (MMPs), which has major impact on the tumor microenvironment (Kessenbrock et al., 2010; Marttila et al., 2014). Our results reconfirm the relationship between *Treponema* and gastric cancer.

*Lactobacillus* is a common probiotic that converts lactose to lactic acid, leading to acidification of the gastric mucosa, which can adapt to growth in gastric juice due to its acidophilic properties (Han et al., 2015). The up-regulation of *Lactobacillus* in GC has been verified in previous studies on gastric mucosa. Interestingly, we found that several differential analyses indicated that *Lactobacillus* presents closely related to the occurrence and development of gastric carcinogenesis. Therefore, the existence of *Lactobacillus* can be regarded as the key bacteria involved in the occurrence of GC. Although *Lactobacillus* acts as a probiotic, in the context of cancer, its metabolite lactic acid can perform an energy source function for tumor cells. It is also a key participant in many carcinogenesis processes, such as metastasis, angiogenesis, metabolism, and immunosuppression (Hirschhaeuser et al., 2011; Hayes et al., 2021). After the Warburg effect was proposed (Warburg et al., 1927), subsequent studies found that lactate, as an immune destroyer of the tumor microenvironment, could directly mediate its effects on cells, such as by blocking cytotoxicity, motility. Its effects can also be indirectly mediated on cells by inducing immunosuppressive cell types such as Tregs, TAMs, and MDSCs. Immune escape driven by lactate within the tumor microenvironment is a major contributor to cancer growth, progression and metastasis (Brand et al., 2016; Corbet and Feron, 2017). In tumor microenvironment, *Lactobacilli* do not end up with the Warburg effect of lactic acid production, but as lactate is continuously released from transformed cells to initiate carcinogenesis in susceptible cells and tissues (Brizel et al., 2001; San-Millan and Brooks, 2017). Therefore, *Lactobacillus* showed an upward trend in the GC group, revealing that it plays an important role in GC. A study using network co-occurrence analysis pointed out that there is an interaction between *Lactobacillus* and other gastric microbiome (Wang et al., 2020). The increase of *Lactobacillus* may lead to ecological dysregulation of the gastric fluid by interaction with other bacteria, thus enhancing the carcinogenic potential of the gastric microbiome. Therefore, we can state that *Lactobacillus* is an important factor for the next studies on gastric cancer prevention and treatment.

Nowadays, a wealth of evidence suggests that changes in the microbiome were related to carcinogenesis. Dysbiosis of the gastric microbiome was reflected not only at the level of changes in the abundance of microbiome members but also in the altered relationships of microbial interactions (Chen et al., 2020; Zhang et al., 2020). Many studies have pointed out that there is widespread competition between bacteria instead of cooperation in networks (Palmer and Foster, 2022). Our network analysis showed that the GPL group had higher network connectivity and complexity and lower aggregation, while the GC group had significantly sparser network connectivity. The high degree of cooperation of GPL group microbial community was closely related to *Cupriavidus*. It was a multifunctional microorganism found in soil and water, which is resistant to heavy metals and has been studied from environmental samples as well as human samples (Vaneechoutte et al., 2004). In previous studies, *Cupriavidus* has been reported associated with invasive human infections, such as bacteremia, pneumonia, immunocompromised patients and cystic fibrosis (*CF*) patients (Coenye et al., 2005; Kobayashi et al., 2016; Bianco et al., 2018). Here, we found that *Cupriavidus* played a key role in the stage of GPL. We hypothesize that *Cupriavidus* was associated with the development of GC. Moreover, Pathogenic bacteria breaching the mucosal protective barrier may make contributions to the decreased aggregation in the GC group, thereby promoting a decrease in the degree of microbial interaction in gastric juice (Krishnan et al., 2020). Compared with HC, the gastric microbiome in the GPL and GC groups changed in composition, ecological network, and function. These changes may be used as relevant factors for predicting carcinogenesis in the future.

In conclusion, our research provides evidence for the imbalance in gastric microbiome in GC patients, clarifies the differences in gastric juice microbiome among HC, GPL, and GC groups, elucidates the changes of gastric microbiome during the development of GC. Our study shows that *Lactobacillus* is an important indicator strain in the course of gastric cancer. The changes in gastric juice microbial community interactions are indicative of the development of gastric cancer. For now, gastric cancer is still a difficult clinical problem. The genus *Lactobacillus* is composed of more than 100 species. The biological behavior of different species varies greatly. At the same time, the interactions between *Lactobacillus* and other microbiome should not be underestimated. Although we propose a key role for *Lactobacillus*, its specific mechanism of action on gastric cancer remains to be further investigated, this will lay the foundation for the study of gastric cancer.

## Data availability statement

The 16S rRNA gene data reported in this paper have been deposited in the Sequence Read Archive (https://www.ncbi.nlm.nih.gov/sra), under accession number PRJNA849572 (https://www.ncbi.nlm.nih.gov/bioproject/PRJNA849572/).

## Ethics statement

The studies involving human participants were reviewed and approved by the independent Ethics Committee of Xiangya Hospital of Central South University following the ethical guidelines of the Declaration of Helsinki (No. 038, 2015). The patients/participants provided their written informed consent to participate in this study.

## Author contributions

LC and ZY designed the experiments. SY carried out experiments. XP analyzed data prepared the figures. XP and SY drafted the manuscript. YZ, HC, and JH participation in discussion and revised the manuscript. All authors contributed to this manuscript, read, and approved the final manuscript.

## Funding

This work was funded by the National Natural Science Foundation of China (32170071 and 32000054).

## Conflict of interest

The authors declare that the research was conducted in the absence of any commercial or financial relationships that could be construed as a potential conflict of interest.

## Publisher’s note

All claims expressed in this article are solely those of the authors and do not necessarily represent those of their affiliated organizations, or those of the publisher, the editors and the reviewers. Any product that may be evaluated in this article, or claim that may be made by its manufacturer, is not guaranteed or endorsed by the publisher.

## Footnotes

^1^https://qiime2.org/

^2^https://gephi.org/

^3^http://huttenhower.sph.harvard.edu/galaxy/
