# Supplementary material for: Alterations in bacterial community dynamics from noncancerous to Gastric cancer
Source: Front Microbiol. 2023 Mar 9;14:1138928. doi: 10.3389/fmicb.2023.1138928 (PMC10034189; doi:10.3389/fmicb.2023.1138928)
Supplement: Supplementary file 1 [file Data_Sheet_1.docx]

Supplementary Material

## Supplementary Figures

**
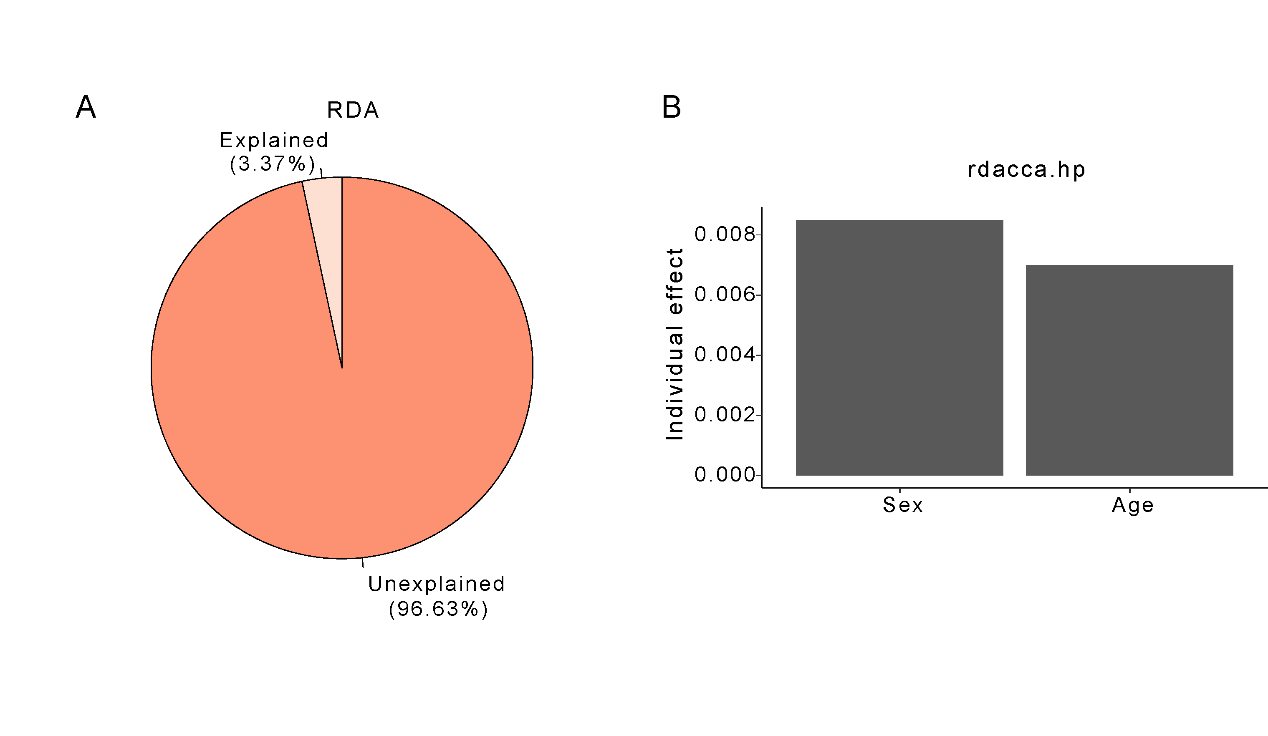
**

**Fig. S1 A.** RDA analysis of sample information and community composition. The pie chart shows the explanation of sample information (Age and Sex) to the bacterial composition and distribution of gastric juice structure. **B.** The Age and Sex individual effect to the bacterial composition and distribution of gastric juice structure base on Hierarchical Partitioning.


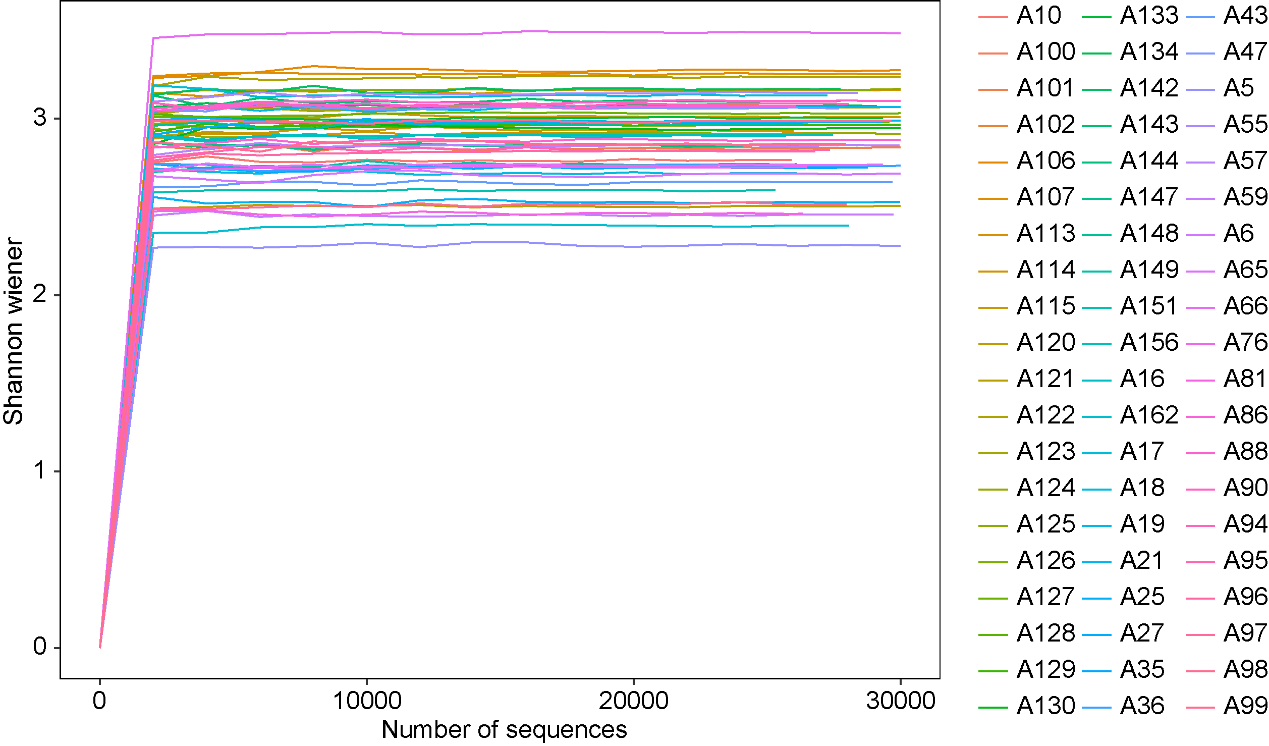


**Fig. S2 Alpha dilution curve for all samples (n = 60).**


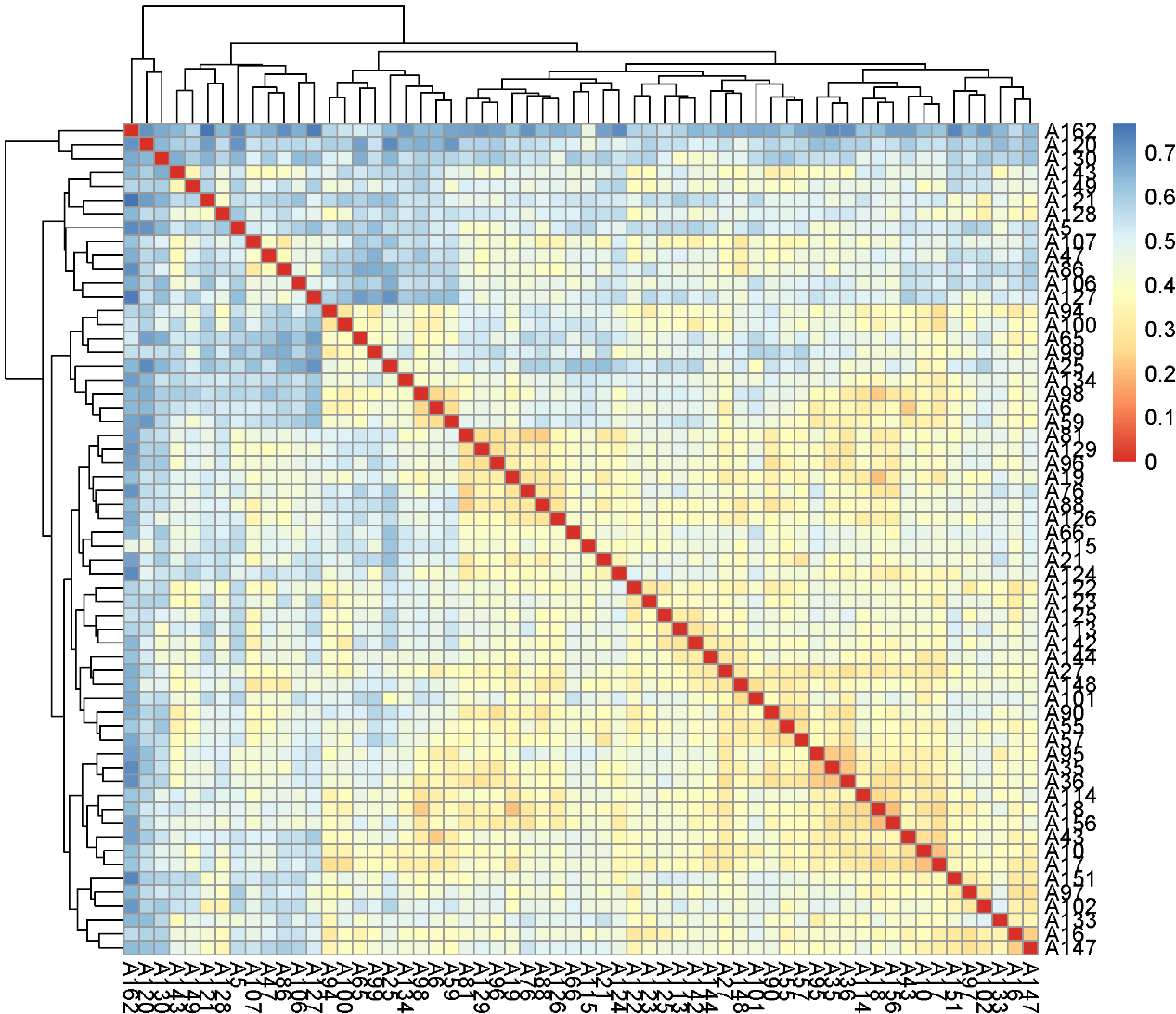


**Fig. S3 Heatmap of bary correlation among samples.**


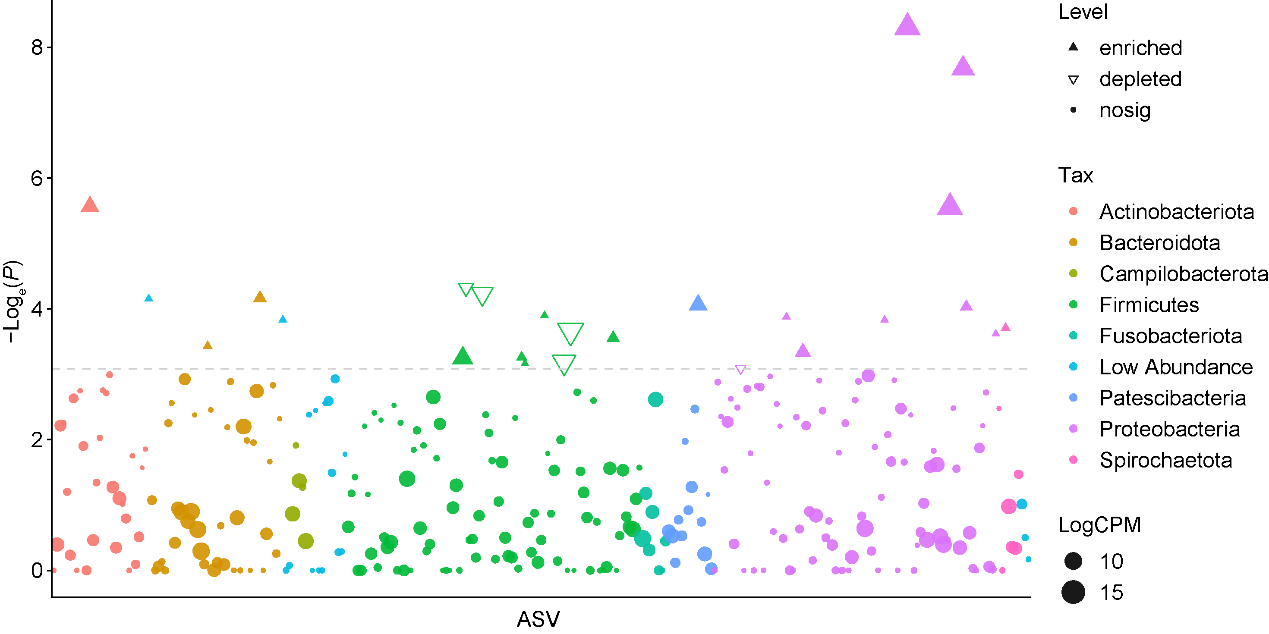


**Fig. S4 Demonstration of gastric cancer (GC) versus bacterial group with gastric precancerous lesions (GPL)**. X-axis for ASVs, alphabetically ordered by taxonomic phylum level; Y-axis *P* value values for the comparison of the two groups, taken as log_e_(*P*), i.e. natural logarithmic transformation; the size of the nodes in the graph represents the relative abundance of that ASV, taken as log_2_(CPM), the logarithm of 2; CPM is an abbreviation for count per million, which being fractions of a million; different nodes colors represent different phylum; the shape of the nodes in the graph marks the type of its change, whether it is up-regulated enriched (positive solid triangle), down-regulated depleted (inverted hollow triangle), or no significant difference change nosig (solid nodes).


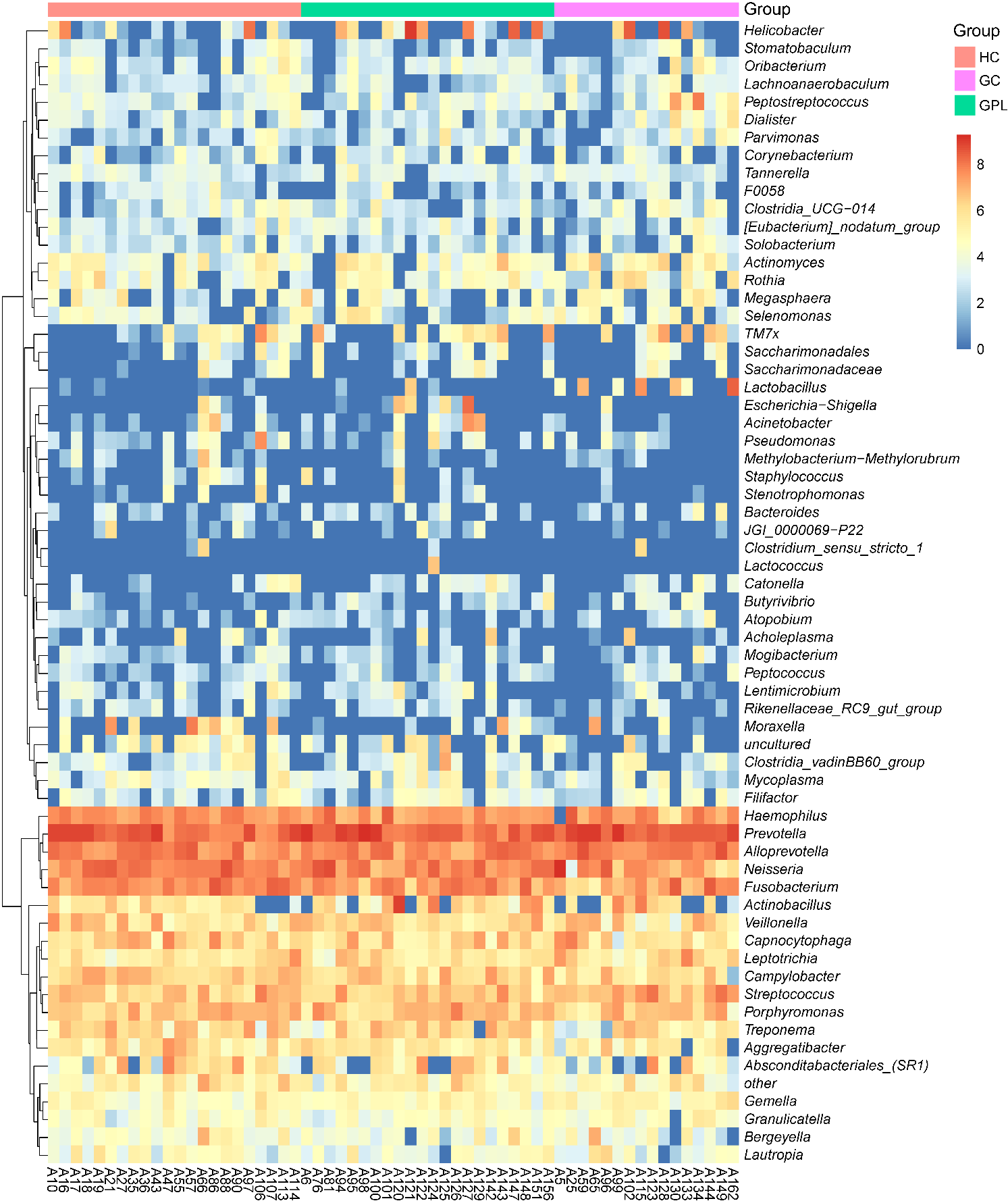


**Fig. S5 Genus Level Abundance Heatmap.** Red indicates high relative abundance, blue indicates low relative abundance. HC, healthy control; GPL, gastric precancerous lesions; GC, gastric cancer.


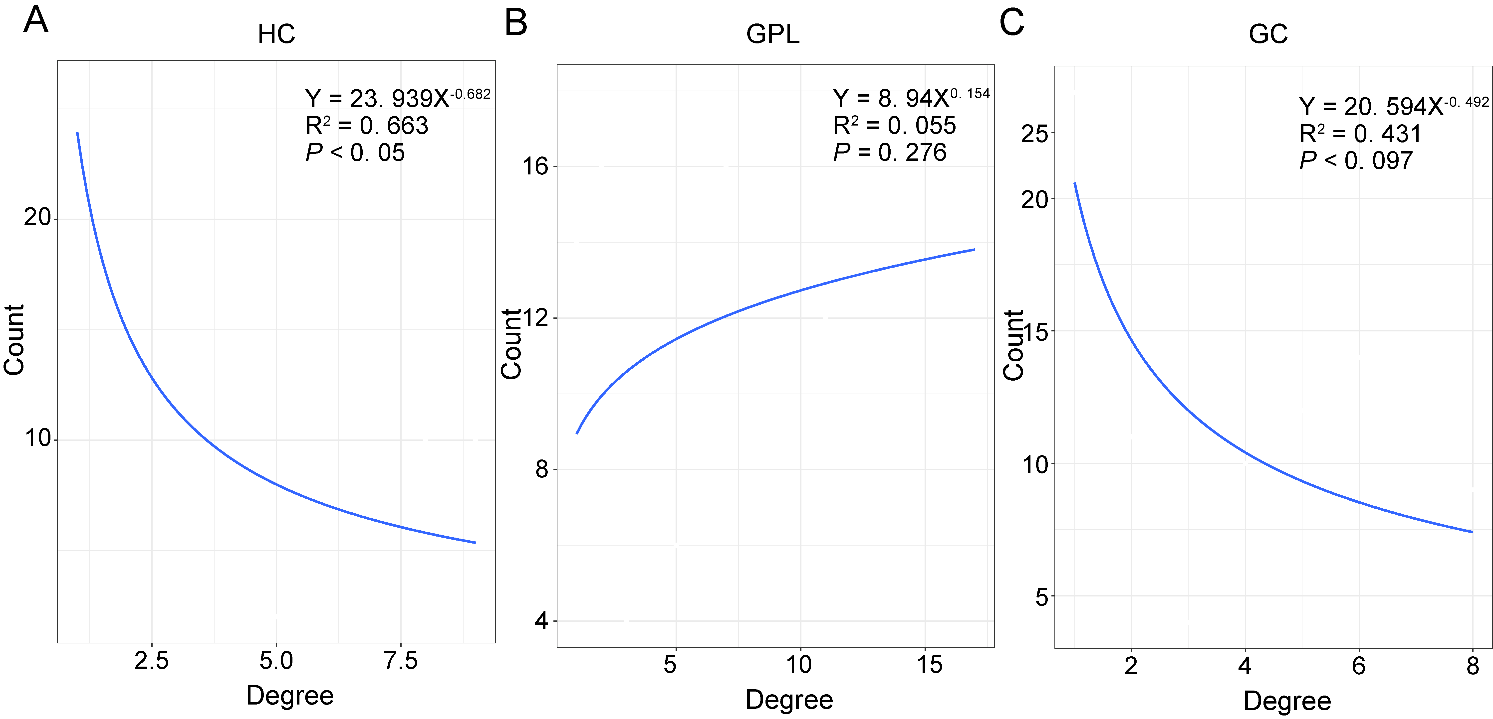


**Fig. S6** **The distributions of degree for the three groups’ real co-occurrence network.** The *P-*value was obtained by randomly permuting the count values 999 times and obtaining the R^2^ (R^2^) from the data after randomly permuting the data. Comparing the frequency that R^2^ of the randomly permuted value is greater than the R^2^ of the observed value, that is, the *P-*value. HC, healthy control; GPL, gastric precancerous lesions; GC, gastric cancer.


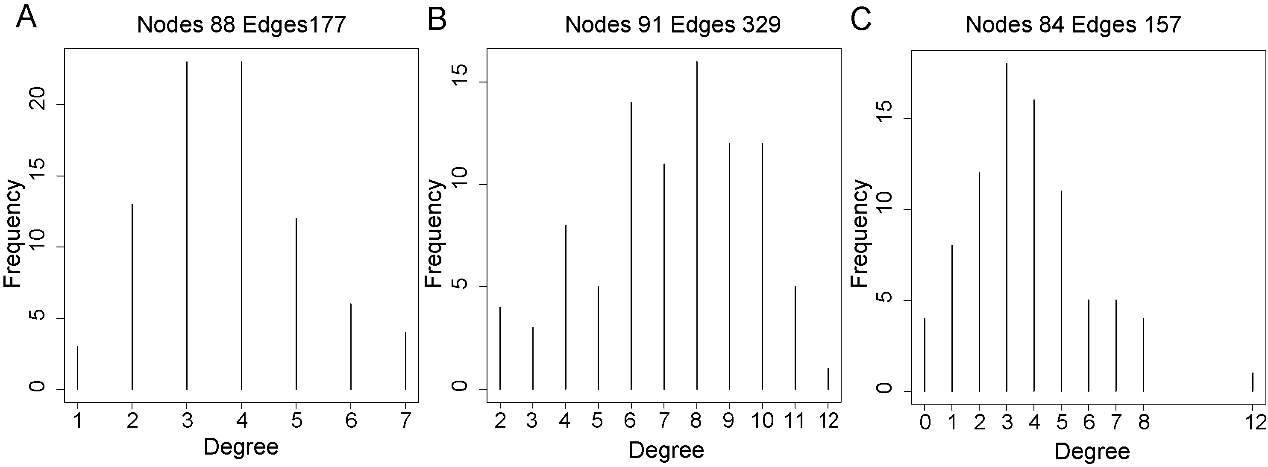


**Fig. S7** **The distributions of degree for the associated random networks.** Same nodes and edges with real co-occurrence network, healthy control **(A)**, gastric precancerous lesions **(B)**, gastric cancer **(C)**.

**Supplementary tables**

| **Helicobacter pylori infection** | **HC**  **(n = 22)** | **GPL**  **(n = 22)** | **GC**  **(n = 16)** |
| --- | --- | --- | --- |
| Positive | 6 | 9 | 11 |
| Negative | 16 | 13 | 5 |

**Table S1 The condition of Helicobacter pylori infection.**

**Table S2** **Helicobacter pylori infection in patients.**

| **Sample ID** | **Group** | **Helicobacter pylori infection** |
| --- | --- | --- |
| A10 | HC | Negative |
| A16 | HC | Positive |
| A17 | HC | Negative |
| A18 | HC | Negative |
| A19 | HC | Negative |
| A21 | HC | Positive |
| A27 | HC | Positive |
| A35 | HC | Negative |
| A36 | HC | Negative |
| A43 | HC | Negative |
| A47 | HC | Negative |
| A55 | HC | Negative |
| A57 | HC | Negative |
| A66 | HC | Positive |
| A86 | HC | Negative |
| A88 | HC | Positive |
| A90 | HC | Negative |
| A97 | HC | Positive |
| A106 | HC | Negative |
| A107 | HC | Negative |
| A113 | HC | Negative |
| A114 | HC | Negative |
| A6 | GPL | Negative |
| A76 | GPL | Positive |
| A81 | GPL | Negative |
| A94 | GPL | Positive |
| A95 | GPL | Positive |
| A98 | GPL | Negative |
| A100 | GPL | Negative |
| A101 | GPL | Negative |
| A120 | GPL | Positive |
| A121 | GPL | Positive |
| A122 | GPL | Positive |
| A124 | GPL | Negative |
| A125 | GPL | Negative |
| A126 | GPL | Negative |
| A127 | GPL | Positive |
| A129 | GPL | Negative |
| A142 | GPL | Negative |
| A143 | GPL | Negative |
| A147 | GPL | Positive |
| A148 | GPL | Negative |
| A151 | GPL | Positive |
| A156 | GPL | Negative |
| A5 | GC | Negative |
| A25 | GC | Positive |
| A59 | GC | Negative |
| A65 | GC | Positive |
| A96 | GC | Positive |
| A99 | GC | Positive |
| A102 | GC | Positive |
| A115 | GC | Negative |
| A123 | GC | Positive |
| A128 | GC | Positive |
| A130 | GC | Negative |
| A133 | GC | Positive |
| A134 | GC | Positive |
| A144 | GC | Negative |
| A149 | GC | Positive |
| A162 | GC | Positive |

**Table S3 The topological features of network**

|  | Nodes ^a^ | Edges ^b^ | Modularity ^c^ | | Average clustering coefficient ^d^ | Network diameter ^e^ | Average path length ^f^ | Average degree ^g^ | | No. of modules |
| --- | --- | --- | --- | --- | --- | --- | --- | --- | --- | --- |
| HC  GPL  GC | 88  91  84 | 177  329  157 | 0. 844  0. 722  0. 879 | 0. 944  0. 983  0. 967 | | 4  2  2 | 1. 243  1. 006  1. 013 | | 3. 738  7. 231  4. 023 | 23  19  23 |

HC, healthy control; GPL, gastric precancerous lesions; GC, gastric cancer.

^a^ Number of OTUs with the correlation *r* > 0. 8 or *r* < -0. 8 and statistical significance (*P* < 0.05)

^b^ Number of strong and significant correlations between nodes

^c^ Modularity > 0.4 suggests that the network has a modular structure. It indicates that there are nodes in the network that are more densely connected between each other than with the rest of the network and that their density is noticeably higher than the graph’s average.

^d^ How nodes are embedded in their neighborhood, and the degree to which nodes tend to cluster together

^e^ The maximum distance between all possible pairs of nodes

^f^ The average number of steps along the shortest paths for all possible pairs of network nodes

^g^ Node connectivity showing how many connections (on average) each node has to the other nodes in the network

| Nodes ^a^ | Edges ^b^ | Modularity (SD) ^c^ | Average clustering coefficient (SD) ^d^ | Average path length (SD) ^e^ | Average degree ^f^ |
| --- | --- | --- | --- | --- | --- |
| 88  91  84 | 177  329  157 | 0. 449±0.002  0. 311±0.001  0. 468±0.015 | 0. 048±0.014  0. 079±0.001  0. 046±0.014 | 3. 303±0.057  2. 476±0.013  3. 412±0.071 | 4. 023  7. 231  3. 738 |

**Table S4 The associated random networks**

Random networks were generated by rewiring all of the links with the same numbers of nodes and edges to the real networks

The number in the brackets indicates the standard deviation of topological properties of the 100 Erdös-Rényi random networks

^a^ Number of the associated random networks nodes

^b^ Number of the associated random networks edges

^c^ Modularity >0.4 suggests that the network has a modular structure. It indicates that there are nodes in the network that are more densely connected between each other than with the rest of the network and that their density is noticeably higher than the graph’s average.

^d^ How nodes are embedded in their neighborhood, and the degree to which nodes tend to cluster together

^e^ The average number of steps along the shortest paths for all possible pairs of network nodes

^f^ Node connectivity showing how many connections (on average) each node has to the other nodes in the network
